# Risk Factors of Forced Take-Off in Dairy Cows Milked Three Times per Day in A Rotary Milking Parlor: A Case Control Study

**DOI:** 10.3390/ani11102883

**Published:** 2021-10-03

**Authors:** Matthias Wieland, Paul Douglas Virkler, Anja Sipka

**Affiliations:** Department of Population Medicine and Diagnostic Sciences, Cornell University, Ithaca, NY 14853, USA; pdv3@cornell.edu (P.D.V.); ass233@cornell.edu (A.S.)

**Keywords:** bovine, forced retract, machine milking, milk yield, on-farm milk meter

## Abstract

**Simple Summary:**

To remain competitive on the global market in the presence of shrinking profit margins, dairy producers continuously seek to increase the efficiency of their operations. One way to increase the operating efficiency in rotary milking parlors is to enhance the rotational speed of the platform. However, increasing the rotational speed reduces the maximum milking duration that is possible during a single rotation. This can increase the number of cows that have not finished milking and milk remaining in the mammary gland if the milking unit is removed early by means of a forced take-off, a practice that is typical for dairy herds in North America and some European countries. To provide a means for monitoring these cows, we described a protocol for the identification of cows that repeatedly are subjected to a forced take-off (RFTO) at the end of milking. Using this protocol, we then investigated cow characteristics that were associated with RFTO. The associations found suggested that the developed protocol could serve as a useful tool to identify cows at risk of RFTO. Through modification of the case definition, dairy producers will be able to adjust their monitoring protocol according to their individual farm-goals.

**Abstract:**

The aims of the research were to: (1) describe a protocol for the identification of cows that are subjected repeatedly to a forced retraction event at the end of milking; (2) study risk factors of repeated forced take-off (RFTO); and (3) assess the average milk flow rate at which the forced retraction event occurred. In a retrospective study, we collected milk flow data over a 1-week period from a 4300-cow dairy with a rotary milking parlor and a thrice-daily milking schedule. We identified 109 cases of RFTO and 2467 controls. A multivariable logistic regression model revealed associations of parity, stage of lactation, average daily milk production, and milking speed with RFTO. Cows in parity 3 or greater, animals ≤100 days in milk, high-producing animals, and cows with low milking speed had higher odds of RFTO. The average (least squares means (95% CI)) milk flow rates at the time of removal of the milking unit were 2.1 (2.0–2.1) kg/min in milking observations that were terminated with the forced retract and 1.5 (1.4–1.5) kg/min when milking units were removed with the automatic cluster remover. Future research to better understand the effect of RFTO on milk production, udder health, and animal well-being is warranted.

## 1. Introduction

One of the single largest expenses on a dairy operation is the milking center [1]. To remain competitive on the global market, dairy farmers therefore continue to pursue opportunities to increase the efficiency of their parlor operations, spreading the capital investment over as many kg of harvested milk as possible. Large dairy operations (≥500 cows) in New Zealand and Australia employ rotary parlor systems to milk their cows [2]. This is consistent with data from the National Animal Health Monitoring System suggesting that US dairy operations “with inventories considerably larger than 500 cows used rotary parlors” [3]. Following the trajectory in the dairy industry with a reduction in total herd numbers and a contemporary increase in the average size of herds in many countries within the European Union and internationally [4,5,6], rotary milking parlors will likely become one of the dominating milking systems [2].

One way to increase the operating efficiency of a rotary milking parlor is to increase the speed of the platform. The rotational or platform speed is measured in s/milking stall, whereas the rotation time is defined as the time needed to complete one rotation. The rotation time can be calculated via multiplication of the rotational speed by the number of milking stalls on the platform [2]. Increasing the rotational speed can increase cow throughput and decrease the idle time of the milking unit (i.e., occupation time of the milking stall without harvesting milk) [2]. Consequently, dairy operations have increased the rotational speed of their rotary milking parlors over the last decades. In 2001, Armstrong et al. [7] suggested a rotational speed of 11–12 s/milking stall. Edwards et al. [2] collected data from 62 New Zealand dairy farms with rotary parlors in 2010 and reported a mean rotational speed of 6.8 s/milking stall in rotary milking parlors that were operated by two milking technicians. This trajectory is consistent with observations from our own group indicating that the rotational speed in rotary parlors is limited only by the ability of the milking technicians completing individual tasks of the pre-milking udder preparation and can be as fast as 5 s/milking stall.

However, a rotational speed of this magnitude can hamper the ability to achieve adequate teat stimulation, impact the accuracy of clinical mastitis detection, and reduce the efficacy of teat sanitization measures (i.e., drying and cleaning of teats). Further, increasing the rotational speed results in a reduction in the maximum milking duration that is possible during a single rotation. This can increase the number of cows that have not finished milking at the end of their rotation. In pasture-based systems, these cows have been allowed a second rotation. Edwards et al. [2] suggested that the optimum percentage of such “go-around” cows would be approximately 20% if a potential “shadow effect” (i.e., the “go-around” cow causes the following milking stall to be unoccupied) was included. A practice to further increase the milking efficiency of a rotary milking parlor and maximize the time that clusters are harvesting milk is to remove the milking unit early through application of a maximum cluster attachment time [8,9]. This regimen has been proposed recently for pasture-based systems [10] and is typical for North American and European operations where it is referred to as “forced retract” or “forced take-off” [11]. However, there is growing concern among dairy operators regarding the amount of milk that remains in the mammary gland in cows that are subjected to a forced retract and its potential impact on milk production, udder health, and animal well-being. The objectives of this study, therefore, were to: (1) describe a protocol for the identification of cows that are subjected repeatedly to a forced retraction event at the end of milking; (2) study risk factors of repeated forced take-off (RFTO); and (3) assess the average milk flow rate at which the forced retraction event occurred.

## 2. Materials and Methods

### 2.1. Data Acquisition

We conducted this retrospective case–control study in July 2021 at a commercial dairy farm located near Ithaca, New York. During the study period, 4221 to 4283 lactating Holstein cows per day were housed in free-stall pens and were bedded with manure solids. Cows were fed a total mixed ration formulated to meet the requirements outlined by the National Research Council [12]. Herd data were maintained in a dairy management software program (Dairy Comp 305, Valley Agricultural Software, Tulare, CA, USA). The farm has used Dairy Herd Improvement Association services including the individual-cow somatic cell count (SCC) option. The average (mean ± standard deviation (SD)) production level during the study period was 42.5 ± 10.4 kg/cow per day.

### 2.2. Milking System

Cows were milked 3 times daily at 01:00, 09:00, and 17:00 h in a 100-stall rotary parlor (RP3100HD, DeLaval International AB, Tumba, Sweden) where cows stand side by side in an 75° angle towards the outer edge and are serviced from the outside of the platform. The vacuum pump (22.4 kW; 30 HP) was regulated by a variable frequency drive and set to supply a receiver operator vacuum of 47.1 kPa (13.9 inHg). The milking unit was composed of the cluster MC70 (DeLaval International AB, Tumba, Sweden) and a milking liner with a round barrel shape (LS-01 NC, DeLaval International AB, Tumba, Sweden). The pulsators (EP100, DeLaval International AB, Tumba, Sweden) were set to a pulsation rate of 60 cycles/min, a ratio of 65:35, and a side-to-side alternating pulsation. The pulsation phases under load were assessed with a vacuum recorder (Tri-Scan, GEA Farm Technologies, Inc., Bönen, Germany). The measurements were a-phase, 152; b-phase, 493; c-phase, 109; and d-phase, 247ms. The average claw vacuum during the peak milk flow period was calculated from 10 milking observations using a vacuum recorder (Tri-Scan, GEA Farm Technologies, Inc., Bönen, Germany) according to the guidelines outlined by the National Mastitis Council [13] and was 38.9 kPa (11.5 inHg). The automatic cluster removers were set to a cluster remover milk flow threshold of 1.5 kg/min, a 1-s delay, and a vacuum decay time of 1.5 s. The milk sweep was initiated 3.0 s after unit retraction and lasted for 2 s. Milking system settings and milking characteristics were monitored with a dairy farm management software program (DelPro, DeLaval International AB, Tumba, Sweden).

### 2.3. Milking Routine 

The rotational speed of the milking parlor was 5.3 s/stall for early- and mid-lactation cows and 4.9 s/stall for late-lactation animals. The parlor was operated by 5 milking technicians who were assigned to the following tasks: Task 1 was to manually forestrip teats and apply pre-milking teat disinfectant with a dip applicator cup. Task 2 was to wipe the teat barrel of all teats from lactating quarters with an individual clean cloth towel. Task 3 was to wipe the teat end with an individual clean cloth towel. Task 4 was to attach and align the milking unit. The positioning of the tasks were (assuming that cow entrance = stall 1): task 1, stall 3; task 2, stall 11; task 3, stall 13; task 4, stall 22 for early- and mid-lactation cows and stall 27 for late-lactation cows. This set up resulted in a dip contact time of 39–42 s, a stimulation time (i.e., duration of manual fore-stripping) of approximately 2 s, and a preparation lag time (i.e., time from first tactile stimulus to milking unit attachment) of approximately 100 s for early- and mid-lactation and 118 s for late-lactation cows. The 5th milking technician was the designated ‘roamer’. Tasks for this position included to support the milking technicians who were assigned to tasks 1–4 and to take a rest. Milking technicians rotated between tasks after each pen to mitigate physical and mental fatigue during the work shift. Two teat spray robots (TSR, DeLaval International AB) were installed at the parlor exit for post-milking teat dip application at positions 92–93 and 94–95 (stalls 96–100: exit area). The forced take-off was initiated at stall 90 resulting in a maximum milking unit-on time of 360 s for early- and mid-lactation animals and 309 s for late-lactation cows.

### 2.4. Data Acquisition

We obtained information on cow characteristics such as parity, stage of lactation (days in milk; DIM), somatic cell score (SCS; calculated according to Ali and Shook [14] as follows: SCS = ln[(SCC/100,000)/ln(2) + 3]), presence of a non-lactating quarter, and average daily milk production during the observation period from the dairy management software program (DairyComp 305). Milking characteristics (milk yield (kg/milking session), average milk flow rate (kg/min), milking unit-on time (s), presence or absence of a forced retract, and the milk flow rate at removal of the milking unit (kg/min)) were assessed at each milking with electronic on-farm milk meters using near-infrared technology (MM27BC, DeLaval International AB, Tumba, Sweden) and recorded using the dairy farm management software (DelPro, DeLaval International AB, Tumba, Sweden). For this study, we created a report in DelPro to automatically record milking characteristics for each milking session and export them to a comma-separated values (csv) file once per day.

### 2.5. Study Design

The observation period was defined as 1 week (24 June to 30 June, 2021). The study population consisted of all lactating cows during this period. In a first step, we merged all 7 reports containing milk flow characteristics for each day in a single Excel file (Microsoft Excel, 2016 version, Microsoft Corp., Redmond, WA, USA). Subsequently, we screened the data for missing and erroneous values by investigating distributions of milking unit-on time (s) and average milk flow rate (kg/min). We removed observations with missing values, outliers, or probable data errors by excluding observations with values of <100 s and >800 s for milking unit-on time and values <0.1 kg/min and >6 kg/min for average milk flow rate as previously described [11]. In the next step, we calculated the frequency distribution of forced retracts for each individual cow to identify cases and controls. We defined a case of RFTO if the forced retract was documented at least 8 times out of 21 possible milking observations. Cows that had no record of forced retract were included as control cows. Only cows with a complete record of 21 milking observations were included in the subsequent analyses. Finally, case and control cows were extracted into a separate file and merged with an Excel file containing data on parity, stage of lactation, SCS, presence of a non-lactating quarter, as well as mean average daily milk production and average milk flow rate during the observation period.

### 2.6. Analytical Approach 

We performed statistical analyses with the software packages JMP (version 14, SAS Institute Inc., Cary, NC) and SAS (version 9.4, SAS Institute Inc.). Because our particular interest was to study if high-producing cows with low milking speed were at risk of RFTO, we created 2 categorical variables. The variable ADP_Q_ (average daily milk production) was based on the interquartile ranges and had 4 levels with Q1, ≤34.9; Q2, 35–42.6; Q3, 42.7–49.4; and Q4, ≥49.5 kg/d. The variable milking speed (MS) was based on the mean average milk flow rate. For this purpose, we fitted a logistic regression model for the dependent variable RFTO with the continuous variable mean average milk flow rate (kg/min) as independent variable. Second, we determined the optimal cut-point value using a receiver operator characteristic (ROC) curve. The area under the curve was 0.79 and the optimal cut-point was determined to be 3.43 kg/min leading to a sensitivity of 0.82 and a 1-specificity of 0.34. Finally, the binary variable MS was discriminated into low, ≤3.43 kg/min and high, >3.43 kg/min.

#### 2.6.1. Risk Factors of Repeated Forced Take-Off

To investigate risk factors of RFTO, we constructed a multivariable logistic regression model. First, we screened all cow characteristics for inclusion into the multivariable model through univariable analysis. For this purpose, univariable logistic regression models were fitted. Presence or absence of RFTO was the dependent variable and the cow characteristics parity (1st, 2nd, and ≥3rd), stage of lactation (≤100, 101–200, and ≥201 DIM), SCS, presence of a non-lactating quarter, ADP_Q_, and MS were the independent variables and tested one at a time. In the second step, we fitted a multivariable logistic regression model. All variables with a *p*-value < 0.20 in univariable analysis were considered in the initial model. Collinearity among eligible variables was assessed by calculating Spearman correlation coefficients. We considered that a coefficient of > |0.60| indicated collinearity. Manual backward elimination was performed until each of the variables had a *p*-value < 0.05 to establish the final model. Biologically relevant two-way interactions between the remaining variables were tested and those with a *p*-value < 0.05 were retained to fit the final model. We used Deviance and Pearson goodness-of-fit statistics to assess the final model fit. Finally, we calculated the adjusted probabilities and 95% confidence intervals (CI) of RFTO for all variables retained in the final model.

#### 2.6.2. Milk Flow Rate at the Time of Removal of the Milking Unit

To assess differences in milk flow rates at the time of removal of the milking unit between milking observations with and without a forced retract, we fitted a general linear mixed model. For this purpose, we used data from the 54,096 milking observations from the 109 cases and 2467 controls over the 7-day observation period. The continuous variable milk flow rate at the time of removal of the milking unit was included as dependent variable and the binary variable presence or absence of a forced retract as independent variable. Cow and day nested within cow were included as random effects to account for the clustered structure of the data. We assessed the assumptions of homoscedasticity and normality of residuals by the inspection of residual plots versus corresponding predicted values and the examination of quantile–quantile residual plots. To satisfy these assumptions, data of the dependent variable milk flow rate at the time of removal of the milking unit was natural-log transformed. The resulting least squares estimates were consequently back transformed and presented as the geometric mean and 95% CI.

## 3. Results

### 3.1. Description of Study Population

We obtained 89979 milking observations from a total of 4339 cows over the 1-week observation period containing 3552 (3.9%) observations with a forced retract. A total of 2603 (2.9%) outliers were removed. A complete record of 21 milking observations was available for 2576 cows that fulfilled the inclusion criteria for case and controls; 539 cows had complete records and between one to seven forced retract events documented and were excluded. A total of 1224 animals had 1 or more missing observations and were removed. Thus, a total of 109 cases of RFTO and 2467 controls were identified. Table 1 shows baseline characteristics and descriptive statistics of case and control cows, respectively. Cows were in their 1st (846, 32.8%), 2nd (800, 31.1%), and 3rd or greater lactation (930, 36.1%), between 1 and 684 DIM (mean ± SD, 173 ± 110), and had a mean SCS of 2.3 ± 1.9. A total of 331 (12.8%) cows had a non-lactating quarter. The mean (±SD) average daily milk production was 42.4 ± 10.4 kg/d (cases, 53.1 ± 10.2; controls, 41.9 ± 10.1 kg/d). The overall mean average milk flow rate over the 7-day observation period was 3.7 ± 0.7 kg/min (cases, 3.0 ± 0.5 kg/min; controls, 3.8 ± 0.7 kg/min). Table 2 shows the frequency distribution of cases and controls stratified by ADP_Q_ and MS.

### 3.2. Risk Factors of Repeated Forced Take-Off

**Figure 1:**
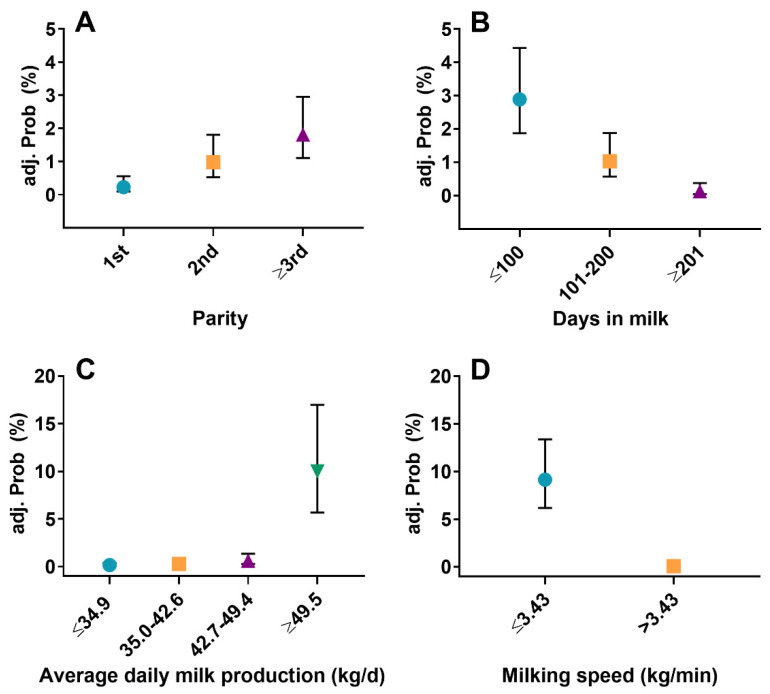
Adjusted probabilities (95% confidence intervals) of repeated forced take-off at the end of machine milking for cows with different parity (**A**), stage of lactation (**B**), average daily milk production (**C**), and milking speed (**D**). (**C**,**D**): error bars partially obscured.

The following independent variables yielded an association with RFTO in univariable analysis: parity (*p* < 0.0001), stage of lactation (*p* < 0.0001), presence or absence of a non-lactating quarter (*p* = 0.05), ADP_Q_ (*p* < 0.0001), and MS (*p* < 0.0001). We detected no collinearity among eligible variables (r ≤ |0.52|). The final multivariable logistic regression model included parity (*p* < 0.0001), stage of lactation (*p* < 0.0001), ADP_Q_ (*p* < 0.0001), and MS (*p* < 0.0001 (Table 3)). Compared with animals in 3rd or greater lactation, the OR (95% CI) of RFTO were 0.12 (0.05–0.30) for cows in parity 1 and 0.54 (0.30–0.96) for cows in parity 2. Cows between 1 and 100 DIM (OR (95% CI): 22.31 (7.81–63.73)) and cows from 101-200 DIM (OR (95% CI): 7.84 (2.68–22.91)) had higher odds of RFTO compared with animals ≥201 DIM. Cows with an average daily milk production of ≥49.5 kg/d had the highest odds RFTO compared with cows that yielded ≤34.9 kg/d (OR (95% CI): 0.01 (0.005–0.04)), between 35 and 42.6 kg/d (OR (95% CI): 0.03 (0.01–0.07)) or between 42.7–49.4 kg/d (OR (95% CI): 0.06 (0.02–0.14)). The odds of RFTO for cows with low MS were higher compared with cows with a high MS (OR (95% CI): 182.50 (79.04–421.39)). None of the tested interactions were retained in the model. Deviance and Pearson goodness-of-fit statistics for the final model revealed *p* = 0.98 and *p* = 0.45. Thus, we accepted the null hypothesis that the model fit the data. The adjusted probabilities (95% CI) of RFTO for cows with different parities, DIM, ADP_Q_, and MS, respectively, are depicted in Figure 1.

### 3.3. Milk Flow Rate at the Time of Removal of the Milking Unit

Among the 54096 milking observations from 109 cases and 2467 controls, 1467 (2.7%) observations were terminated with the forced retract. The frequency distribution between milking sessions was session 1:471 (2.6%); session 2:515 (2.9%); session 3:481 (2.7%). The average (mean ± SD) milk flow rate at the time of removal of the milking unit was 2.3 ± 0.6 kg/min for observations that were terminated with the forced retract and 1.5 ± 0.2 kg/min for observations that were terminated using the automatic cluster removal. Least squares means (95% CI) from the general linear mixed model for milking observations with and without forced retract, respectively, were 2.1 (2.0–2.1) kg/min and 1.5 (1.4–1.5) kg/min (*p* ˂ 0.0001). The assumptions of homoscedasticity and normality of residuals were met.

## 4. Discussion

### 4.1. Identification of Cows with Repeated Forced Take-Off

Here, we describe a protocol for the identification of cows with RFTO using data from electronic on-farm milk meters. Our work extends the existing literature aimed to improve the knowledge and enhance the sustainability of the milk harvesting process [2,11,15]. The idea for this study arose through our extension service activities with dairy producers who ask for help identifying cows with RFTO. Such knowledge would allow dairy producers to identify cows whose maximum milk-production capacity may not have been harvested with the current parlor settings and target cows who may benefit the most from a more laborious and cost-intensive milking regimen. This may facilitate efficient use of their parlor and labor resources. Through modification of the case definition presented here, dairy producers can adjust their monitoring protocol according to their individual farm-goals.

### 4.2. Risk Factors of Repeated Forced Take-Off

We found an association between average daily milk production and RFTO such that cows with an ADP_Q_ ≥ 49.5 kg/d had higher odds of RFTO. Similarly, we observed that cows with low milking speed (i.e., MS ≤ 3.43 kg/min) had higher odds of RFTO.

Parity was associated with RFTO with cows in 3rd or greater lactation having higher odds of RFTO compared with animals in 1st or 2nd lactation. Last, cows with ≤100 DIM had higher odds of RFTO compared with animals between 101–200 DIM and cows with ≥201 DIM. These findings are most likely attributable to differences in milking unit-on time between cows with different parity and at different stages of lactation, as cows in parity 3 or greater and early lactation animals have longer milking durations [16].

No associations were found for SCS and presence of a non-lactating quarter. Based on a recent study [16] showing that milking unit-on time in cows with a non-lactating quarter was longer compared with cows with 4 lactating quarters, we had expected an association between presence of a non-lactating quarter and RFTO. The absence thereof is likely due to a contemporary decrease in milk production in cows with a non-lactating quarter [16], which likely offset the prolonging effect of presence of a non-lactating quarter on milking unit-on time.

### 4.3. Milk Flow Rate at the Time of Removal of the Milking Unit

An extension of our study was to investigate at what milk flow rate the forced retraction event occurred to form an idea of how much milk is likely to remain in the mammary gland of cows subjected to a forced retraction event. Our results show that the average milk flow rate was 2.1 kg/min when milking units were removed via forced retraction. One may differentiate two potential negative effects of incomplete milking on milk production. First, the immediate loss in milk yield due to incomplete milk harvest resulting in milk remaining in the mammary gland. Second, the medium-term and long-term effects of milk remaining in the mammary gland through autocrine–paracrine factors that modulate the secretion, proliferation, and apoptosis of mammary epithelial cells and affect the milk production rate. In a recent study, Penry et al. [17] enrolled 12 cows with a twice daily milking schedule using a half-udder design to investigate the effect of milk remaining in the mammary gland after milking on milk production rate. The average milk remaining in the mammary gland was approximately 23% of the total half-udder volume. The authors reported an average decrease in milk production rate of approximately 25% over the 6-week study period [17]. Martin et al. [18] utilized the negative effect of incomplete milking on milk synthesis to lower milk production of high yielding cows milked twice per day before dry-off through automated early removal of the milking cluster. For this purpose, the researchers employed a software module that was informed by the cows’ individual milk yield prior to enrollment, which was then reduced by 5% on a daily basis over a duration of 10.4 ± 1.8 days to achieve a gradual milk yield reduction [18]. They reported a daily milk yield reduction of 0.7 kg/day in 26 cows that were subjected to early removal of the milking cluster compared with 0.07 kg/day in 30 control cows for which the milking cluster was removed at a milk flow rate of 0.3 kg/min [18]. The average milk flow rate over the period of incomplete milking in cows subjected to early cluster removal was 3.7 ± 1.2 kg/min [18], which is as high as the milk flow rate between 60–120 s after start of milking in well stimulated cows reported in a recent study [19] and higher than the average milk flow rate when milking units were removed via the forced retraction in the current study. Taken together, these results suggest that in the current study, the average milking observation subjected to a forced retract was terminated during the decline phase of the milk flow curve. We therefore speculate that if an impact of RFTO on milk production in the current study population existed, it would have been minor.

### 4.4. Study Limitations, Practical Application, and Future Directions

Our study had some limitations that the reader should consider. We conducted this study on one commercial dairy farm in New York with high-producing Holstein dairy cows that are milked three times per day in a rotary parlor system. Thus, our results likely reflect commercial operations in this area. However, the external validity of this study may be extrapolated only to similar operations with rotary parlor systems in this region. Some mention should be made of the case definition for RFTO in the current study. The threshold of at least eight milking observations with a forced retract was somewhat arbitrary. Our reasoning was that cows that are subjected to a forced retract in more than one third of milking observations warrant further investigation. However, although the observed relationships between cow characteristics and RFTO were biologically coherent, their biological and economical relevance remained obscure.

Possible approaches to mitigate forced retraction events in a rotary parlor system could be to allow cows a second rotation, a common practice that is used in block-calving, pasture-based systems [11]. Edwards et al. [2] analyzed data from 376,429 milking observations from 44,530 cows from 62 commercial dairy farms in New Zealand and suggested that approximately 20% of “go-around” cows was the optimum to achieve improved parlor throughput. However, “go-around” cows will likely finish milking relatively early during their second rotation causing either idle time of the occupied stall when units are removed automatically, and no milk is being harvested, or excessive overmilking of cows if milking units are removed manually at the end of the rotation. Further, this practice is not typical in North America and Europe [11]. Indeed, in the current study population, the parlor system was not equipped to confine cows in the milking stalls at the end of their rotation. Another option could be to group cows identified with RFTO and adjust the rotational speed accordingly. This would allow harvesting their full milk-production capacity while maintaining parlor efficiency for the remainder of the milking herd. Last, through integration of data from sensor and non-sensor applications that support cow identification, real-time assessment of milk yield and flow rate, and cow characteristics, cows at risk of RFTO on the platform could be identified and inform a variable speed drive controlling the rotational speed of the rotary parlor. In the presented study herd, farm management decided against a modification of the parlor settings but to continue monitoring the frequency distribution of cases of RFTO and milk flow rate at the time of removal of the milking unit with future modifications of their milking center. Dairy operators with different farm-goals may elect to accommodate cows with low MS and adjust their milking routine accordingly.

Future research is needed to further our understanding about the biological and economic impacts of RFTO. Studies should be conducted to validate, and if needed modify, the case definition developed and used in this study. Although we attempted to gather information on how much milk remained in the mammary gland in cows subjected to forced retraction, our results remain speculative. Thus, future studies that include data from multiple dairy operations should examine the effect of RFTO on milk production. The presence of residual milk that remains in the mammary gland after milking has been thought to be associated with decreased udder health. This theory has been supported by findings from Penry et al. [17], who observed an increase in SCC from 26,300 to 48,300 cells/mL when approximately 23% of the total half-udder volume remained in the mammary gland. Future studies should therefore investigate the effect of RFTO on SCC and incidence of clinical mastitis. In this study, we failed to assess the possible effect that RFTO may have on animal well-being. Recently, researchers employed measurements of heart rate variability, step, and rumination behavior to study cows’ stress response to the milking process in a rotary milking system [20]. This work could be extended to examine the effect of RFTO on animal well-being, which is a growing concern of both dairy operators and the consuming public. Such studies should also consider the inter-relationship between different rotational speeds, its influence on the quality of pre-milking udder preparation and RFTO.

## 5. Conclusions

We described a protocol for the identification of cows with RFTO using data from electronic on-farm milk meters. Dairy operators can modify and employ this protocol according to their farm-goals to target cows whose maximum milk-production capacity may not have been harvested with their current milking routine. This would allow them to use their parlor and labor resources most efficiently. The associations between cow characteristics and RFTO documented in this study indicated that high-producing cows and cows with low milking speed have higher odds of RFTO. Using the milk flow rate at the time of removal of the milking unit, we suggested that the average milking observation that was subjected to the forced retract was terminated during the decline phase of the milk flow curve. Future research to better understand the effect of RFTO on milk production, udder health, animal well-being, and farm economics is warranted.

## Figures and Tables

**Table 1:** Baseline characteristics of dairy cows with (cases, n = 109) and without (controls, n = 2467) repeated forced take-off. *p*-values derived from univariable logistic regression analyses testing the association between the respective baseline characteristic and the presence or absence of repeated forced take-off. Note percentage may not add up to 100 due to rounding error.

Item	Cases	Controls	Overall	*p-*Value
Parity (n, %)				<0.0001
1st	8 (0.3)	838 (32.5)	846 (32.8)	
2nd	36 (1.4)	764 (29.7)	800 (31.1)	
≥3rd	65 (2.5)	865 (33.6)	930 (36.1)	
Stage of lactation (DIM)				<0.0001
≤100	69 (2.7)	695 (27.0)	764 (29.7)	
101–200	35 (1.4)	770 (29.9)	805 (31.3)	
≥201	5 (0.2)	1002 (38.9)	1007 (39.1)	
Somatic cell score (mean ± SD)	2.2 ± 2.0	2.3 ± 1.9	2.3 ± 1.9	0.73
Non-lactating quarter (n, %)				0.05
Present	7 (0.3)	324 (12.6)	331 (12.9)	
Absent	102 (4.0)	2143 (83.2)	2245 (87.2)	
Average milk production (kg/d)				<0.0001
≤34.9	8 (0.3)	643 (25.0)	651 (25.3)	
35–42.6	12 (0.5)	644 (25.0)	656 (25.5)	
42.7–49.4	16 (0.6)	624 (24.2)	640 (24.8)	
≥49.5	73 (2.8)	556 (21.6)	629 (24.4)	
Milking speed (kg/min)				<0.0001
≤3.43	87 (3.4)	827 (32.1)	914 (35.5)	
>3.43	22 (0.9)	1640 (63.7)	1662 (64.5)	

**Table 2:** Frequency distribution of dairy cows with (cases, n = 109) and without (controls, n = 2,467) repeated forced take-off stratified by average daily milk production categorized into 4 levels based on interquartile ranges (Q1, ≤34.9; Q2, 35–42.6; Q3, 42.7–49.4; and Q4, ≥49.5 kg/d) and milking speed (MS) categorized into 2 levels (low, ≤3.43 and high, >3.43 kg/min).

	Average Daily Milk Production								
MS	Cases				Controls				Total
	Q1	Q2	Q3	Q4	Q1	Q2	Q3	Q4	
≤3.43	8 (7.3)	12 (11.0)	16 (14.7)	51 (46.8)	469 (19.0)	249 (10.1)	99 (4.0)	10 (0.4)	914
>3.43	0 (0)	0 (0)	0 (0)	22 (20.2)	174 (7.1)	395 (16.0)	525 (21.3)	546 (22.1)	1662
Total	8 (7.3)	12 (11.0)	16 (14.7)	73 (67.0)	643 (26.1)	644 (26.1)	624 (25.3)	556 (22.5)	2576

**Table 3:** Results of multivariable logistic regression model showing the association of parity, stage of lactation, average daily milk production, and milking speed with repeated forced take-off at the end of machine milking.

Item	ß^2^ (SE)	*p*-Value	aOR^3^ (95% CI)
Parity		<0.0001	
1st	−2.09 (0.46)		0.12 (0.05–0.30)
2nd	−0.62 (0.30)		0.54 (0.30–0.96)
≥3rd	Referent		-
Stage of lactation (DIM)		<0.0001	
≤100	3.10 (0.54)		22.31 (7.81–63.73)
101–200	2.06 (0.55)		7.84 (2.68–22.91)
≥201	Referent		-
Average daily milk yield (kg/d)		<0.0001	
≤34.9	−4.27 (0.54)		0.01 (0.005–0.04)
35–42.6	−3.66 (0.49)		0.03 (0.01–0.07)
42.7–49.4	−2.87 (0.45)		0.06 (0.02–0.14)
≥49.5	Referent		-
Milking speed (kg/min)		<0.0001	
≤3.43	5.21 (0.43)		182.50 (79.04–421.39)
>3.43	Referent		-

## Data Availability

The data presented in this study are available on request from the corresponding author.
